# Efficacy and Safety of BCG Vaccine for Control of Tuberculosis in Domestic Livestock and Wildlife

**DOI:** 10.3389/fvets.2018.00259

**Published:** 2018-10-26

**Authors:** Bryce M. Buddle, Hans Martin Vordermeier, Mark A. Chambers, Lin-Mari de Klerk-Lorist

**Affiliations:** ^1^AgResearch, Hopkirk Research Institute, Palmerston North, New Zealand; ^2^Animal and Plant Health Agency, Addlestone, United Kingdom; ^3^Faculty of Health & Medical Sciences, School of Veterinary Medicine, University of Surrey, Guildford, United Kingdom; ^4^Veterinary Wildlife Services, Kruger National Park, Department of Agriculture, Forestry and Fisheries, Pretoria, South Africa

**Keywords:** BCG, cattle, diagnosis, goats, deer, tuberculosis, vaccination, wildlife

## Abstract

Bovine tuberculosis (TB) continues to be an intractable problem in many countries, particularly where “test and slaughter” policies cannot be implemented or where wildlife reservoirs of *Mycobacterium bovis* infection serve as a recurrent source of infection for domestic livestock. Alternative control measures are urgently required and vaccination is a promising option. Although the *M. bovis* bacille Calmette-Guérin (BCG) vaccine has been used in humans for nearly a century, its use in animals has been limited, principally as protection against TB has been incomplete and vaccination may result in animals reacting in the tuberculin skin test. Valuable insights have been gained over the past 25 years to optimise protection induced by BCG vaccine in animals and in the development of tests to differentiate infected from vaccinated animals (DIVA). This review examines factors affecting the efficacy of BCG vaccine in cattle, recent field trials, use of DIVA tests and the effectiveness of BCG vaccine in other domestic livestock as well as in wildlife. Oral delivery of BCG vaccine to wildlife reservoirs of infection such as European badgers, brushtail possums, wild boar, and deer has been shown to induce protection against TB and could prove to be a practical means to vaccinate these species at scale. Testing of BCG vaccine in a wide range of animal species has indicated that it is safe and vaccination has the potential to be a valuable tool to assist in the control of TB in both domestic livestock and wildlife.

## Introduction

Tuberculosis (TB) in domestic livestock and wildlife is caused by *Mycobacterium bovis, Mycobacterium caprae*, and other members of the *Mycobacterium tuberculosis* complex, including *M. tuberculosis* whose role in animal TB is being increasingly recognised, particularly in studies from Africa and Asia (1). Livestock TB continues to be a major economic animal health problem worldwide. It has been estimated that >50 million cattle are infected worldwide, costing US$3 billion annually (2). The disease is an important zoonosis, causing TB in humans, particularly through consumption of unpasteurised milk or through co-habitation with infected animals. The “test and slaughter” bovine TB control programmes introduced in many countries in the mid-twentieth century achieved dramatic results and a number of countries were able to eradicate this disease. However, these control programmes have not been affordable or socially acceptable in many developing countries, and more than 94% of the world's population live in countries in which control of TB in cattle or buffaloes is limited or absent (3). Furthermore, a confounding factor in the control of bovine TB in a number of countries has been the existence of wildlife reservoirs of *M. bovis* infection.

Wildlife serving as maintenance hosts for *M. bovis* include the Australian brushtail possum (*Trichosurus vulpecula*) in New Zealand, the European badger (*Meles meles*) in United Kingdom (UK) and Ireland, white-tailed deer (*Odocoileus virginianus*) in Michigan, USA [reviewed in (4)] and Eurasian wild boar (*Sus scrofa*) in the Iberian Peninsula, Spain (5). In addition, red deer (*Cervus elaphus*) in several parts of Europe (6), African buffalo (*Syncerus caffer*) in South Africa (7), and wood bison (*Bison bison athabascae*) and wapiti (*Cervus elephus manitobensis*) in Canada (8) serve as maintenance hosts for infection in hunting estates and national parks. These various maintenance hosts act as sources of infection for domestic species, and in national parks, infection can spill over to other unique wildlife species including Iberian lynx (*Lynx pardinus*), lions (*Panthera leo*), leopard (*Panthera pardus*), and wild dogs (*Lycaon pictus*). Partial control has been achieved for some of these maintenance hosts by minimising contact with livestock, reducing the density of animals or banning artificial feeding that causes local high densities of animals (9–11). However, few if any of these control measures can be implemented for some protected species or where interference of a natural regulated ecosystem is deemed undesirable. For these reasons, the development and use of vaccines for control of TB in both domestic livestock and wild animals is very appealing.

Although no TB vaccines are currently registered for protection against TB in domestic livestock, there is renewed interest in their use from the realisation of the financial impact of bovine TB on animal health and trade, and due to the difficulty controlling the disease. In addition, the use of vaccines to control the TB in wildlife reservoirs of infection could be very valuable in limiting the spread of infection to domestic livestock and *M. bovis* bacille Calmette-Guérin (BCG) was registered for intramuscular administration to badgers in the UK in 2010. Evidence of the use of a vaccine to control a disease in wildlife has been shown from the success of using vaccination to control rabies in foxes in Europe (12). BCG vaccine is the only registered TB vaccine for humans and was developed by Calmette and Guérin from a strain of *M. bovis* originally isolated by Nocard from a case of tuberculous mastitis. Following serial passage of the strain on ox bile glycerine-potato medium for 230 passages, between 1908 and 1919, this variant strain was shown to be attenuated in animals and conferred resistance to challenge of animals with virulent *M. bovis* and *M. tuberculosis* [reviewed in (13)]. The strain was distributed to many countries in the 1920s and continuing passage in differing conditions produced a considerable number of daughter strains, with varying antigenic profiles (14).

Vaccination of humans commenced in 1921 and in a meta-analysis, vaccination of newborns and infants significantly reduced the risk of TB by an average of over 50%, although efficacy ranged from 0 to 80% (15). Many field trials of BCG vaccination of cattle were conducted in the first half of the twentieth century and the major caveats that restricted the use of TB vaccines in cattle were that protection was not complete and vaccination could sensitise animals to respond in traditional TB diagnostic tests. These problems can now be potentially overcome by using vaccination integrated with other control measures and use of diagnostic tests which can differentiate infected from vaccinated animals (DIVA tests). Currently, there is very large effort to develop improved TB vaccines for humans, by developing vaccines which may replace BCG or those that could boost immunity following initial vaccination with BCG (16). Research to develop improved TB vaccines for livestock is following a similar path. Information on the efficacy of BCG in animals can be of assistance in the development of these new generation vaccines for use in multiple species. For most wildlife species, however, vaccination efforts are restricted to the use of a single-shot vaccine since access to the same individuals in order to deliver a booster is unrealistic. The focus of this paper is to provide a review of the efficacy and safety of BCG vaccine in domestic livestock and wildlife to assist in optimising the use of BCG vaccine in animals as well as providing a guide for the development of improved TB vaccines. TB vaccines that do not use BCG are being studied for some applications, such as a heat-inactivated *M. bovis* whole-cell vaccine for wild boar, but these are not the focus of this review.

## Vaccination of cattle

### Historical studies of BCG

Studies of BCG vaccine in cattle were first reported by Calmette and Guérin (17) and showed that relatively high doses of BCG (20 mg) could induce protection in cattle against experimental challenge with *M. bovis*. In the studies of Calmette and Guérin (18), intravenous challenge of control calves with virulent *M. bovis* resulted in severe generalised TB by 30–60 days. In contrast, the BCG vaccinates which were challenged remained healthy, but virulent *M. bovis* could be isolated from their bronchial lymph nodes when the animals were killed at 3–4 months post-challenge. A number of other researchers reported similar results in experimental challenge studies where BCG vaccination did not induce absolute immunity, but moderated the severity of the infection [reviewed in (19)]. A comparison of routes of vaccination with BCG showed that intravenous, intradermal and oral routes conferred some resistance to feeding milk containing large doses of virulent *M. bovis*, although not greater than that conferred with subcutaneous vaccination (20). Field studies of BCG vaccination of cattle using either a subcutaneous or intravenous route of vaccination showed variable results which may in part have been influenced by the duration and the potency of the exposure. Promising results were shown by Rankin (21) with 86% (37 of 43) non-vaccinates with tuberculous lesions compared to 33% (22 of 66) for the BCG vaccinates by 8–10 months post exposure. Watson (22) undertook a study over a longer duration where new-born calves were vaccinated with BCG subcutaneously (50–100 mg dose), fed pasteurised milk for 1–2 months, and then exposed to *M. bovis* through ingestion of raw milk from infected cows or co-habitation with infected cattle. The study demonstrated that there was good resistance in calves compared to controls by 1 year post-exposure, but resistance declined steadily up to reproductive age where there was little evidence of protection. A number of uncontrolled trials were undertaken to determine whether BCG vaccination could clear TB infection in heavily infected herds. Some studies reported that BCG vaccination eliminated disease over 7 years (23, 24), while others found this approach reduced the skin test reactivity and resulted in newly introduced unvaccinated animals (*n* = 100) remaining skin test negative over a 5 year post-vaccination period. This approach was judged to be impractical and slow (25).

These early studies indicated that BCG could induce some protection against TB, although protection was not absolute, appeared to wane after 1–2 years and vaccination could induce positive reactivity in the tuberculin test. It was concluded that TB could be eradicated faster and more efficiently using “test and slaughter” control programmes than relying only on vaccination with BCG. However, it was considered that BCG vaccination could possibly have a role in disease control in countries where “test and slaughter” programmes could not be implemented due to economic or social reasons and a number of trials were conducted in Malawi in the 1970s for this purpose. Ellwood and Waddington (26) showed that the development of tuberculous lesions and progressive infection was less in BCG vaccinates following experimental *M. bovis* challenge compared to controls, providing encouragement to proceed with a field study. In the field trial, 3–12 month old calves were injected with 10^7^ colony forming units (CFU) of BCG (Glaxo strain) and revaccinated 6 months later, while alternate calves in each herd were sham inoculated (27). When the animals were slaughtered and necropsied 5 years after the commencement of study, no significant differences could be found in the number of animals with tuberculous lesions, 36 of 204 (17.7%) in the vaccinates and 44 of 210 (21.0%) in the controls. The numbers of cattle which were bacteriologically positive and those with lesions at more than one site were also similar for the two groups.

Possible reasons for the failure to protect in the field trials could include administration of high doses of BCG (1–100 mg parenterally), very high level of *M. bovis* exposure, exposure of young calves to *M. bovis* through consumption of milk from infected cows prior to vaccination, lack of long-term protection, and prior sensitisation to environmental mycobacteria or helminths. In relation to the dose of BCG, Griffin et al. (28) demonstrated in deer that parenteral vaccination of a high dose of BCG (10^8^ CFU) was less effective than doses of 10^4^-10^7^ CFU BCG, whereas higher doses of BCG in badgers appearred more efficacious (29). This suggests the optimal dose of BCG to use in any given species will probably need to be determined empirically. Informative meta-analysis of field trials in cattle have not been undertaken due to varying doses, vaccination routes and strains of BCG used, together with different methods to measure protection and varying levels of exposure to *M. bovis*. Furthermore, robustly controlled designs and statistical analyses of results were rarely undertaken and in most studies, the vaccinated and non-vaccinated animals were kept on separate farms.

### Recent studies to assess factors affecting BCG vaccine efficacy in cattle

Over the past 25 years, a large number of vaccination/challenge trials have been undertaken in cattle using harmonised models, allowing comparisons between varying studies with BCG tested alone or in comparative studies with other vaccines. Challenge models have focused on using a relatively low challenge dose of *M. bovis* (10^3^-10^4^ CFU) administered via endobronchial/intratracheal inoculation or by aerosol (30, 31). This has resulted in the development of tuberculous lesions mimicking those from the natural disease in the lower respiratory tract. Similar BCG strains have been used (initially Pasteur, then BCG Danish 1331) and protection assessed by quantitative gross, histopathological, and microbiological findings.

It is important to determine factors which influence the efficacy of BCG to optimise the use of the vaccine and Table 1 summarises many of these factors. Results from a number of studies have shown that doses of 10^4^-10^6^ CFU of BCG administered parenterally induced equivalent protection (30, 32), while higher doses (10^8^ CFU) were required to induce protection when BCG was administered orally (33, 34). When BCG vaccine was administered at optimal doses, protection induced by the subcutaneous or oral route was very similar, although an advantage from oral administration of BCG was slightly lower tuberculin skin test reactivity. Combinations of BCG by parenteral and mucosal routes has provided mixed results with no enhancement of protection observed when BCG was administered subcutaneously and orally on the same day (35), but a small enhancement in protection with simultaneous administration of BCG by subcutaneous and endobronchial routes (36). Pasteur and Danish strains of BCG induced similar protection, although the kinetics of the cellular immune response varied with the two strains (37, 38). Calves vaccinated subcutaneously with the Phipps strain of BCG had lower mean rank for the total number of tuberculous lesions following a high challenge dose of *M. bovis* (10^5^ CFU) delivered by aerosol compared to controls, although this difference was not significant (46). Neonatal or very young calves were protected at least as well as older calves (39, 40). In one study, natural pre-sensitisation to environmental mycobacteria appeared to have an adverse effect on subsequent immunity induced by BCG vaccine, with less protection induced compared to that for two other attenuated *M. bovis* vaccines (41). While in another study, there was evidence that *M. avium* exposure induced partial protection against *M. bovis* infection, which could possibly mask subsequent immunity induced by BCG (42). Studies in guinea pigs and mice have provided additional information on the effects of pre-sensitisation with environmental mycobacteria where some strains of *M. avium* masked or blocked any protective effect induced by BCG vaccination, while other strains had no effect (47, 48). Studies in Northern Ireland indicated that co-infection of cattle with a liver fluke, *Fasciola hepatica*, and BCG resulted in a suppression of Th1 type immune responses to BCG, potentially affecting immunity induced by BCG vaccination (49). Vaccination of cattle with BCG 3 weeks after an experimental challenge with *M. bovis*, did not produce a beneficial effect, nor increased tuberculous pathology (45). Protection against experimental challenge was shown to be effective at ≤12 months post-vaccination, but had waned by 24 months post-vaccination (43). Together these studies suggest that immunity wanes between 1 and 2 years post-vaccination when protection is measured in a stringent *M. bovis* challenge model.

**Table 1:** Experimental challenge studies to assess factors affecting the efficacy of BCG vaccine for protection of cattle against TB.

**Factor**	**Age of vaccination**		**BCG strain**	**Vaccine dose (CFU)**	**Vaccine route**	***M. bovis*** **challenge**	**Measure of disease^†^**	**Assessment**	**References**
								**BCG vs. Control**	
Dose of BCG	1.	5–6 months	Pasteur	6 × 10^4^	S/C	I/T	Proportion with lesions	2/15 vs. 10/16^*^	(30)
		5–6 months	Pasteur	6 × 10^6^	S/C	I/T	Proportion with lesions	4/15 vs. 10/16^*^	
	2.	5–6 months	Danish	2 × 10^5^	S/C	E/B	Proportion with lung lesions	3/9 vs. 10/10^*^	(32)
		5–6 months	Danish	2 × 10^6^	S/C	E/B	Proportion with lung lesions	2/9 vs. 10/10^*^	
	3.	6 months	Danish	2 × 10^7^	Oral	I/T	Median total lesion score	5 (0, 8)^‡^vs. 11 (8, 13.5)^*^	(33)
		6 months	Danish	2 × 10^8^	Oral	I/T	Median total lesion score	3.5 (0, 10.5) vs. 11 (8, 13.5)^*^	
	4.	6 months	Danish	1 × 10^6^	Oral	I/T	Median LN lesion score	4 (1, 12) vs. 3.5 (0, 12)	(34)
		6 months	Danish	1 × 10^7^	Oral	I/T	Median LN lesion score	4 (0, 12) vs. 3.5 (0, 12)	
		6 months	Danish	2 × 10^8^	Oral	I/T	Median LN lesion score	1 (0, 9) vs. 3.5 (0, 12)^*^	
Route(s) of vaccination	1.	6 months	Danish	1 × 10^6^	S/C	I/T	Median total lesion score	0 (0, 10) vs. 11 (8, 13.5)^*^	(33)
		6 months	Danish	2 × 10^8^	Oral	I/T	Median total lesion score	3.5 (0, 10.5) vs. 11 (8, 13.5)^*^	
	2.	6 months	Pasteur	10^6^ + 10^9^	S/C + Oral	I/T	Median total lesion score	1 (0, 12) vs. 8 (0, 15)^*^	(35)
		6 months	Pasteur	10^6^	S/C	I/T	Median total lesion score	1 (0, 8) vs. 8 (0, 15)^*^	
		6 months	Pasteur	10^9^	Oral	I/T	Median total lesion score	0.5 (0, 7) vs. 8 (0, 15)^*^	
	3.	5–7 months	Danish	5 × 10^5^ + 5 × 10^5^	S/C + E/B	E/B	Median total lesion score	1 (0, 15) vs. 12 (0, 28)^*^	(36)
		5–7 months	Danish	1 × 10^6^	S/C	E/B	Median total lesion score	3 (0, 15) vs. 12 (0, 28)	
		5–7 months	Danish	1 × 10^6^	E/B	E/B	Median total lesion score	1 (0, 27) vs. 12 (0, 28)	
Strain of BCG	1.	6 months	Pasteur	10^6^	S/C	I/T	Median total lesion score	6 (0, 21) vs. 13.5(4, 17)^*^	(37)
		6 months	Danish	10^6^	S/C	I/T	Median total lesion score	7 (0, 13) vs. 13.5(4, 17)^*^	
	2.	6 months	Pasteur	2 × 10^6^	S/C	I/T	Median total lesion score	2 (0, 18) vs. 16 (10, 20)^*^	(38)
		6 months	Danish	2 × 10^6^	S/C	I/T	Median total lesion score	8 (4, 11) vs. 16 (10, 20)^*^	
Neonatal vaccination	1.	8 h	Pasteur	10^6^	S/C	I/T	Proportion with lesions	0/10 vs. 10/10^*^	(39)
		6 weeks	Pasteur	10^6^	S/C	I/T	Proportion with lesions	1/9 vs. 10/10^*^	
	2.	1 day	Danish	10^6^	S/C	I/N	Median total lesion score	2 (0, 4) vs. 13 (0, 30)^*^	(40)
Pre-sensitisation with *M. avium* spp.	1	5–6 months	Pasteur	10^5^	S/C	I/T	Proportion with LN lesions	14/18 vs. 7/9	(41)
		5–6 months	Atten.1 *M. bovis*	10^6^	S/C	I/T	Proportion with LN lesions	3/9 vs. 7/9^*^	
		5–6 months	Atten.2 *M. bovis*	10^6^	S/C	I/T	Proportion with LN lesions	3/9 vs. 7/9^*^	
	2	4–6 months	*M. avium*	10^6^	S/C	I/N	Median total lesion score	4 (0, 9) vs. 9 (0, 15)	(42)
Duration of protection and revaccination	1.	2–4 weeks	Danish	10^6^	S/C	E/B (12 mths)	Median total lesion score	8 (0, 16) vs. 16 (0, 38)^*^	(43)
						E/B (24 mths)	Median total lesion score	8 (5, 17) vs. 10 (8, 17)	
	2.	2–4 weeks	Danish	3 × 10^5^	S/C	E/B (2.5 yrs)	Median total lesion score	7.5 (0, 17) vs. 10 (3, 17)	(44)
		2–4 weeks and 2 years	Danish	3 × 10^5^	S/C	E/B (2.5 yrs)	Median total lesion score	4 (0, 10) vs. 10 (3, 17)^*^	
Vaccination pre- or post-challenge	1.	5–6 months	Danish	1 × 10^6^ (pre-challenge)	S/C	E/B	Proportion with LN lesions	4/12 vs. 10/12^*^	(45)
				1 × 10^6^ (3wks post-challenge)	S/C	E/B	Proportion with LN lesions	11/12 vs. 10/12	

† Measure of protection was standardized where possible and in some cases, recalculated from original data.

‡ Median (range);

* P < 0.05.

CFU, Colony Forming Unit; S/C, Subcutaneous; I/T, Intratracheal; E/B, Endobronchial; I/N, Intranasal.

*Atten. 1 M. bovis and Atten. 2 M. bovis: Two attenuated strains of M. bovis*.

Two studies report the effect of revaccination with BCG. In the first study, calves vaccinated within 8 h of birth or at 6 weeks of age showed a high level of protection against *M. bovis*, while those vaccinated within 8 h of birth and revaccinated at 6 weeks of age had reduced protection (39). The revaccinated calves with the lowest level of protection had the strongest antigen-specific IFN-γ responses post-initial vaccination, suggesting that revaccination had induced an inappropriate immune response. In neonatal calves, antigen-specific IFN-γ responses remain at elevated levels for longer than those seen in older calves, possibly due to a more active BCG infection and BCG revaccination of young calves may be contra-indicated. In contrast, calves vaccinated with BCG at 2–4 weeks of age and revaccinated at 2 years of age when immunity had waned, showed a significant level of protection when challenged 6 months later, while those receiving only the initial vaccine dose were not protected when challenged at the same time (44).

In the past decade a number of field BCG vaccination trials or experiments have been undertaken under natural transmission (in contact) conditions and have provided insights into the effectiveness of BCG vaccine under different levels of disease prevalence over varying time periods (Table 2). The field experiments in Mexico (50) and Ethiopia (51, 52) involved the exposure of vaccinated and non-vaccinated calves to herds of cows which had reactor rates of 40% in the Mexican experiment and 100% in the two experiments in Ethiopia. In the Mexican experiment, vaccination induced a significant level of protection against TB and the vaccine efficacy was estimated to be 59.4%. The level of exposure in the experiments in Ethiopia was very high with ~85% of the non-vaccinated calves developing tuberculous lesions. Despite this high level of exposure, the vaccine efficacy in the first experiment was considered to be similar to that in the Mexican experiment and there were significantly fewer vaccinated animals with lesions and culture positive for *M. bovis* as well as significantly more vaccinated animals that would have passed slaughterhouse meat inspection than that for the controls (51). The vaccine efficacy in the second experiment conducted in Ethiopia when measured by comparing lesioned, culture or histology-positive animals in the BCG-vaccinated group with naïve controls was relatively low (around 30%) (52). However, in this last experiment, the severity of pathology and dissemination of *M. bovis* was significantly lower in the vaccinated, infected animals compared to that for the non-vaccinated animals, which could relate to a lower ability to transmit disease (onward transmission). The difference between the two Ethiopian experiments was attributed to a higher prevalence of overt clinical signs of TB in the infected herd in the second experiment.

**Table 2:** Recent field trials to assess the efficacy of BCG for protection of cattle against TB.

**Trial**	**Country**	**BCG strain**	**Vaccine dose**	**Vaccine route**	**Age at vaccination**	**Source of infection**	**In-contact (exposure) time**	**Measurement of disease**	**Assessment**	**References**
									**BCG vs. Control**	
1.	Mexico	Tokyo	10^6^	S/C	1–2 weeks	Infected herd (40% herd reactor prevalence)	10 months	Proportion positive in three tests: PPD skin test, PPD IFN-γ test, ESAT-6/CFP10 IFN-γ test	6/65 vs. 15/66^*^ (9.2% vs. 22.8%) 59.4% efficacy	(50)
2.	Ethopia	Danish	10^6^	S/C	2 weeks	Infected herd (100% herd reactor prevalence)	10–23 months	Proportion with lesions	5/13 vs. 12/14^*^	(51)
									(39% vs. 86%)	
								Mean total pathology score (95% CI)	4.6 vs. 14.1^*^	
									(0, 10.5) vs. (2.5, 24.6)	
3.	Ethopia	Danish	10^6^	S/C	2 weeks	Infected herd (100% herd reactor prevalence)	1 year	Proportion with lesions	15/23 vs. 22/26	(52)
									(65% vs. 85%)	
								Mean total pathology score (95% CI)	4.0 (1.8, 6.1) vs. 7.8^*^ 2.5, 13.1)	
4.	New Zealand	Danish	10^8^	Oral	6–30 months	Infected herd and wildlife (5–10% herd reactor prevalence)	1–4 years	Proportion with TB lesions and/or *M. bovis* cultured	31/644 vs. 63/531^*^	(53)
									(4.8% vs. 11.9%)	
									67.4% efficacy	

CFU, Colony Forming Units; S/C, Subcutaneous; CI Confidence interval; IFN-γ, Gamma-interferon; PPD, Purified protein derivative

* *P < 0.05*.

A large field trial was undertaken in New Zealand to evaluate the efficacy of BCG vaccine administered orally (53). Free-ranging, vaccinated and non-vaccinated cattle were stocked at low densities and were naturally exposed to *M. bovis* for periods of 1–4 years from tuberculin reactor cattle (reactor herd prevalence of 5–10%) and a wildlife reservoir of infection (brushtail possums). BCG vaccine was administered orally to cattle in an attempt to reduce tuberculin skin test reactivity. This trial included 1,286 cattle and at slaughter the prevalence of infection was 4.8% among vaccinates and 11.9% in non-vaccinates. The overall vaccine efficacy was estimated to be 67.4%, but higher for those killed within 2 years post-vaccination (77.4%). Vaccination also appeared to slow the progression of TB, with infected vaccinates more likely to have no visible lesions and less likely to have a high lesion score.

In summary, the field experiments and trials have shown that BCG vaccination can markedly reduce the number of cattle infected with *M. bovis*, which is different to that seen in the experimental challenge trials where vaccination only reduced the severity of the disease. However, an exception was in the field trial when there was a very high exposure to *M. bovis* (52). With the longer exposure periods, there appeared to be a waning of immunity after 2 years (53).

### Differentiating infected from vaccinated animals (DIVA) tests

It is well-established that vaccination with BCG can compromise the interpretation of the tuberculin skin test, which serves as the primary surveillance test for “test and slaughter” bovine TB control strategies. Using the single intradermal comparative cervical test, 80% of BCG-vaccinated calves were shown to react in the tuberculin skin test at 6 months post-vaccination, but decreasing to 10–20% by 9 months post-vaccination (54) and in a another study, the maximum skin test reactivity was observed after 5 weeks, but disappeared completely by 18 months after vaccination (55). Positive responses were also observed in the caudal fold skin test at 6 months after BCG vaccination compared to that for a corresponding control group, but there were no differences between the groups by 12 months after vaccination (44). DIVA tests will be required for countries intending to use BCG vaccination alongside conventional “test and slaughter” control strategies. DIVA tests have now been developed using antigens from the *M. tuberculosis* complex which are not expressed or secreted by BCG and can be used instead of bovine PPD in the whole blood IFN-γ or skin tests. Two of the antigens used in the DIVA tests are the ESAT-6 and CFP10 proteins, which are encoded in the RD1 region of *M. tuberculosis* and *M. bovis*, but not in BCG, which has lost this region of its genome (56–58), and a third antigen, Rv3615c, which is not located in the RD1 region, but its secretion is dependent on the esx-1 secretion system located in the RD1 region (59). A recent evaluation of the whole blood IFN-γ test incorporating ESAT-6, CFP10, and Rv3615c indicated that the sensitivity was similar to that with the comparative tuberculin readout using avian and bovine PPD. When tested in non-infected animals, both the DIVA and tuberculin readouts gave similar specificities of between 97 and 99%. The relative specificity of the DIVA blood test was also high (95%) in BCG-vaccinated cattle and was significantly greater than that observed for the tuberculin readout (71%) (60). One scenario to use the DIVA blood test would be to re-test tuberculin-positive cattle; alternatively, it is also possible to use these antigens in a skin test rather than the IFN-γ test. The DIVA skin test in cattle has now been shown to have a high sensitivity for *M. bovis*-infected cattle, to a similar level than that for the comparative cervical skin test in non-vaccinated cattle while not compromised by vaccination with BCG or with vaccines against Johne's disease (61, 62).

## Vaccination of goats

TB infection of goats is caused by *M. bovis* or *M. caprae* and in the natural disease lesions are predominantly found in the lungs and associated lymph nodes, indicating an aerosol route of infection (63). The disease is responsible for economic losses in endemic areas and infected goats may be a source of TB for cattle or humans. Caprine TB is present in a number of European countries, but currently there are no caprine TB control campaigns in the European Union. To determine protective efficacy of vaccines, gross and microscopic lesions have been assessed by qualitative and quantitative analyses, together with mycobacterial culture from lung-associated lymph nodes. The precise determination of the total lung lesion burden related to total lung volume has been achieved using multi-detector computed tomography (64).

BCG Danish vaccine administered subcutaneously at a dose of 5 × 10^5^ CFU was shown to be safe and no shedding of BCG was detected in the faeces of vaccinated kids or in the milk of vaccinated, lactating goats (65). BCG was isolated from a lymph node draining the site of vaccination from one kid at 8 weeks post-vaccination, but not from any goats at 24 weeks post-vaccination. A single dose of BCG vaccine administered subcutaneously to goats was shown to significantly induce protection against an endobronchial challenge with *M. caprae*, with reductions in pulmonary pathology and bacterial load. Vaccination with BCG appeared to prevent haematogenous dissemination of mycobacteria with extra-thoracic TB lesions only found in non-vaccinated goats (66). A comparison of parenterally administered BCG and heat-inactivated *M. bovis* vaccines showed that both vaccines provided similar levels of protection against a *M. caprae* experimental challenge, with a reduction in the volume of thoracic TB lesions and extra-pulmonary lesions compared to non-vaccinates (67). Use of mycobacterial DIVA reagents, ESAT-6 and CFP10, in the IFN-γ test was able to differentiate TB-infected from BCG-vaccinated goats. A field BCG vaccination trial was recently undertaken in a herd of goats infected with *M. caprae* (68). Twenty-three goat kids were vaccinated subcutaneously with 10^5^ CFU of BCG Danish, with a further 22 kids serving as non-vaccinated controls. Two months later, the kids were mixed with a herd of goats which had a TB reactor rate of 79%. Sixteen months later, all trial goats were killed and necropsied. Vaccination significantly reduced the number of animals with TB lesions compared to that for non-vaccinates (35 and 77% respectively; representing a vaccine efficacy of 53%) and when extrapulmonary cases were considered, the reduction were even higher (17 and 68%, respectively; vaccine efficacy of 75%). Vaccination has been seen as a valuable long-term control prospect, reducing the TB prevalence prior to starting a test and slaughter eradication programme which would reduce economic costs for producers and the public sector.

## Vaccination of sheep

Sheep have traditional been considered a rare host for the *M. tuberculosis* complex, but can be part of a multi-species system which may maintain TB in a region, at least in mixed farms where sheep cohabit with TB-infected cattle and/or goats (69). In a trial where lambs were vaccinated parenterally with BCG Danish and subsequently challenged endobronchially with *M. caprae*, the vaccinated lambs had a significant reduction in gross lesions compared to the non-vaccinated controls (70). All challenged lambs developed gross lesions in the respiratory system, which were similar to those observed in goats experimentally challenged with *M. caprae* at a similar dose.

## Vaccination of deer

TB in farmed and feral deer is predominantly caused by *M. bovis*, and in the USA and Spain, feral deer also serve as a wildlife reservoir of *M. bovis* infection, acting as a source of infection for domestic livestock (6, 10). Deer serve as important domestic livestock species, farmed predominantly for the production of venison, while feral deer are valued for hunting. Tuberculous lesions are commonly described as liquefied or abscess-like in contrast to the caseous nature of the lesions seen in cattle and goats (71, 72). The most frequent site of the tuberculous lesions is in the retropharyngeal lymph nodes, followed by lesions in the lungs and associated lymph nodes as well as in the mesenteric lymph nodes (73). BCG vaccination studies of deer has been undertaken to assess whether vaccination could be an effective method of protecting farmed deer from TB and in feral deer to prevent reinfection back into cattle herds.

Studies of BCG vaccine in red deer have shown that a single dose of BCG administered subcutaneously to 3 month old deer could reduce disease severity, while revaccinating deer at intervals of 8–16 weeks intervals induced protection against infection, but not at an interval of 43 weeks (74). Increasing the time period between booster dose and *M. bovis* challenge from 6 to 26 or 52 weeks had no significant effect on protection. Two doses of 10^4^-10^7^ CFU of BCG induced protection against TB, but less with a dose of 10^8^ CFU and killed BCG in a mineral-in-oil adjuvant induced no protection (28). A study in red deer in Spain compared oral administration of BCG Danish (10^8^ CFU) with oral administration of heat-inactivated *M. bovis*, 10^7^ bacilli, followed by an experimental challenge with *M. bovis* (75). Only the heat-inactivated vaccine induced a significant reduction in lesion pathology compared to that for the non-vaccinates, however, the results were constrained by very small group sizes (5 animals/group). Neither vaccine induced a bovine PPD IFN-γ response post-vaccination. Parenteral BCG administered at a dose of 10^6^ CFU or oral BCG at 10^8^ CFU induced a similar degree of protection in white-tailed deer (76). Parenteral vaccination with either BCG Danish or Pasteur resulted in decreased disease severity, without sterile immunity (77). A booster dose 6 weeks later did not raise the level of protection (78). BCG was shown to persist for 3–9 months in lymphoid tissues of deer vaccinated parenterally or orally (79). Evidence has been provided of transmission of BCG from parenterally vaccinated deer to in-contact, non-vaccinated deer (77, 79). In another study, deer orally vaccinated with 10^9^ CFU BCG Danish were housed with non-vaccinated deer for 27 weeks. There was immunological evidence of transmission of BCG to the non-vaccinated animals, but no BCG could be isolated from the tissues of either group of animals when killed 27 weeks after vaccination (80). There was no evidence (immunologically or by culture) of transmission of BCG to the cattle which were exposed to the room previously occupied by the vaccinated deer. Complications can occur with the delivery of oral vaccine baits to feral deer as the provision of supplementary feed to feral deer can lead to large numbers of deer congregating together resulting in the spread of TB (81), also there are concerns about non-target uptake of live vaccine baits, particularly by cattle. Simulation modelling has examined the potential role that vaccination could play in control programmes to minimise cattle herd breakdowns (82). Vaccination of 50–90% of susceptible deer within a 5 km radius of cattle farms was predicted to result in a 95% probability of having no cattle herd breakdowns in 15–18 years.

## BCG vaccination of wildlife

The requirements for a vaccine for wildlife differ to those for domestic livestock in that preferentially, the vaccine would be self-administered via an oral route and animals would only receive a single vaccination. Vaccination should prevent the spread of infection to other wildlife or livestock, but complete protection against infection would not be necessary. Recent studies in multiple wildlife species have shown that BCG vaccine can fulfil these requirements and provide protection against TB (Table 3).

**Table 3:** Summary of BCG vaccine efficacy studies in wildlife.

**Species/Country**	**Route^†^**	**Challenge type**	**Vaccine efficacy^‡^**	**Notes/Particular issues**	**Key references**
Bushtail possum/New Zealand	O,M, P	Aerosol,	+	High vaccine cost compared to that for poisons	(83)
		natural exposure	+		(84)
European badger/ UK, Ireland	O,M, P	Endobronchial, Natural exposure	+ +	Parenteral vaccine licensed (BadgerBCG). For an oral vaccine: demonstration of consistent protection	(29, 85) (86–88)
White-tailed deer/ USA	O,P	Intratonsilar	+	BCG persistence in tissues, bans on supplementary baiting, non-target bait uptake	(76, 77)
Eurasian wild boar/Spain	O	Oral	+	Non-target bait uptake	(89)
		Natural exposure	±	Regulatory issues	(90)
Ferret/New Zealand	O,P	Oral	±	Rarely maintenance host for *M. bovis*	(91, 92)
African buffalo/ South Africa	P	Intratonsilar	–	Practicality of vaccine delivery in the field	(7)

† Vaccination route, O, oral; M, other mucosal; P, parenteral.

‡ *Vaccine efficacy, + protection, ± partial protection, – no protection*.

### Vaccination of brushtail possums

The brushtail possum is the major wildlife reservoir of *M. bovis* infection in New Zealand as well as declared as a noxious pest. Possums are highly susceptible to *M. bovis* infection and lesions are found predominantly in the lungs and superficial lymph nodes. Culling of possums by trapping and poisoning has been a major contributor to the dramatic reduction in the numbers of infected cattle over the past 20 years (11). Vaccination of possums against TB has the potential to be an effective TB control measure when it is not suitable to cull possums such as near urban areas.

BCG vaccination of possums via a number of different routes including subcutaneous, intranasal and oral have induced a significant level of protection against experimental *M. bovis* challenge by intratracheal and aerosol routes (83, 93). Oral administration of BCG via baits would be the preferred route of administration of BCG vaccine to wild possums, but it was shown that direct administration of BCG intragastrically was less effective compared to administered by the same route and mixed with a drug to reduce gastric acidity or when administered intraduodenally (94, 95). To increase the efficacy of oral administered BCG vaccine, the BCG bacilli were encapsulated in a lipid matrix which protected the bacteria from degradation in the acidic stomach environment, resulting in improved protection against a *M. bovis* challenge as well as increasing shelf life of the vaccine in the field (83, 96). Vaccine-induced immunity was shown to wane between 6 and 12 months post-vaccination following oral vaccination and there were no differences between BCG doses of 10^7^ and 10^8^ CFU or between Danish and Pasteur strains of BCG (97). A more recent study indicated that protection against an experimental *M. bovis* infection extended out to 28 months post-vaccination (98). BCG bacilli were shown to be stable in the lipid matrix for 7 weeks under room temperature conditions and 3–5 weeks under field conditions in a forest/pasture habitat, when maintained in weather-proof, bait-delivery sachets. Furthermore, uptake of oral bait placebo vaccines was shown to be high with 85–100% of wild possums accessing baits at bait densities of 40–80 sachets/hectare (96). Possums consuming oral bait BCG vaccine, containing 10^8^ CFU of BCG, displayed no adverse clinical signs, but shed relatively low concentrations of BCG in their faeces, 10^2^-10^4^ CFU/g faeces, for up to a week and BCG could be isolated from their mesenteric lymph nodes for up to 8 weeks post-vaccination (99).

Two field trials have been undertaken in possums to determine efficacy of BCG vaccine against natural exposure to *M. bovis* infection. In the first trial, BCG vaccine was administered intranasally and intraconjunctivally (total dose of 10^6^ CFU of BCG Pasteur) to possums trapped in the field, with an equivalent number left non-vaccinated. After vaccination, the animals were released back into the field site, which was endemic for TB in wildlife (100). The animals were trapped, examined for clinical TB and released again every 2 months. Two years after the start of the study, possums were recaptured, killed and examined for TB lesions. Vaccination significantly reduced the proportion of possums infected with *M. bovis* (4/149 for vaccinates and 13/151 for non-vaccinates), with a vaccine efficacy of 69% for prevention of TB. The second field study was of a similar design, but with BCG vaccine administered orally in a lipid matrix (total dose 10^8^ CFU BCG Danish). Again, there was a significant reduction in the proportion of infected possums in vaccinates (1/51) compared to that for the non-vaccinates (12/71), with a vaccine efficacy of 95% for prevention of TB (84). In contrast to the experimental challenge studies, protection against natural exposure to *M. bovis* vaccination resulted in protection against infection. The major constraint for the use of BCG vaccine in possums in New Zealand is cost of the vaccine compared to that for poisons, particularly when possums are considered as a noxious pest.

### Vaccination of badgers

The European badger is the major wildlife reservoir of *M. bovis* infection in Great Britain due to their relative abundance and ecology, the prevalence of infection and presentation of TB pathology compared to other sylvatic species (101, 102). Options for preventing the transmission of *M. bovis* from infected badgers to cattle are limited to minimising the potential for contact between them (biosecurity), reducing the number and density of infected badgers via selective and non-selective culling, and vaccination [reviewed in (103)]. Badgers are protected by law in the UK and Ireland which limits the public acceptability and practicality of culling and for disease control, and culling of badgers in England and Ireland has sometimes delivered conflicting results that likely reflect subtle differences in the epidemiology of the disease locally (104). Vaccination of badgers against TB has the potential to be an effective TB control measure, especially in combination with other control measures (105) and considerable progress has been made in testing the efficacy of BCG vaccine in badgers.

BCG vaccine has been administered to badgers via a variety of routes, including subcutaneous, intramuscular and mucosal (conjunctival and oral) and vaccination by all these routes has induced significant protection against experimental endobronchial challenge with *M. bovis* [reviewed in (103)]. The use of BCG to vaccinate badgers against TB in the UK by the intramuscular route was licensed by the UK Competent Authority (Veterinary Medicines Directorate) in 2010 as BadgerBCG and is available for use by veterinarians and trained lay vaccinators under prescription from a veterinarian. Licensing of BadgerBCG required evidence of vaccine safety and efficacy and laboratory and field studies showed that vaccination of badgers by injection with BCG was both safe and significantly reduced lesions of TB caused by *M. bovis* (29, 106). Protection was incomplete, in that *M. bovis* infection of vaccinated badgers still produced either visible pathology or *M. bovis* was isolated from organs at necropsy. Results from a 4-year field study of BCG in wild badgers were consistent with the direct protective effect of BCG observed in experimental studies. Individual badgers that initially tested negative to a panel of diagnostic tests, presumed uninfected, were significantly less likely to subsequently test positive to serological and immunological tests for TB following vaccination, compared to non-vaccinated control animals (86, 107). Furthermore, non-vaccinated cubs captured in vaccinated social groups were significantly less likely to test positive to TB when more members of their group had been previously vaccinated. The most plausible explanation for this result is that BCG had caused a herd immunity effect, with the rate of *M. bovis* transmission being more effectively reduced in social groups where a higher proportion of animals had been vaccinated.

A practical limitation to the extensive use of BadgerBCG is the need to trap badgers before the vaccine can be injected and the use of an oral bait delivery system would be advantageous. BCG has been incorporated in a wide variety of baits, including encapsulation in the same lipid matrix used to deliver BCG orally to possums. Administration of BCG orally to captive badgers, either directly to the back of the throat, or indirectly via ingested bait has been shown to protect badgers against experimental challenge with *M. bovis* and there was no difference in the levels of protection induced by Pasteur and Danish sub-strains of BCG (85, 108). To assess the vaccine safety, badgers were orally dosed with 10^9^ CFU of BCG, followed 14 days later by a single oral dose of 10^7^ CFU BCG (109). No adverse physical effects were observed, nor effects on the social behaviour and feeding habits of the vaccinated animals. BCG was cultured from the faeces of two of nine vaccinated animals (10^2^ CFU/g) ~48 h after the higher dose of BCG was administered and by one of the nine vaccinated animal (80 CFU/g) ~24 h after receiving the lower dose of BCG. No evidence was found for the transmission of BCG to non-vaccinated, sentinel, badgers housed with the vaccinated animals despite the occasional excretion of BCG in faeces. The target dose of BCG for the oral vaccination of badgers is yet to be defined.

A field trial was recently completed in Ireland that provided the first estimate of oral BCG efficacy under field conditions (87). Lipid-encapsulated BCG was delivered to the back of the throat of anaesthetised badgers, whilst other badgers received only the lipid as placebo. The study area was divided into three equally representative zones with different proportions (0, 50, and 100%) of the badger population in each zone being vaccinated with either BCG or placebo. Attempts were made to capture badgers every 6 months and between the first two capture periods the vaccine efficacy was estimated to be 36%, while it was 84% for capture periods 3–6. Among the vaccinated badgers that seroconverted, the median time to seroconversion (413 days) was significantly longer when compared with non-vaccinated animals (230 days). In addition, there was a significant reduction in the proportion of animals presenting with *M. bovis* culture confirmed lesions in the fully (100%) vaccinated zone (9%), compared with the non-vaccinated (0%) zone (26%).

### Vaccination of wild boar

Wild boar serve as the main wildlife reservoir of the *M. tuberculosis* complex (MTC) in the Mediterranean regions of the Iberian Peninsula, Spain and TB prevalence in wild boar has been associated with TB occurrence on cattle farms (110, 111). Wild boar are widespread in Eurasia and can be found in high densities, particularly on hunting estates (112). These animals are highly susceptible to MTC infection and lesions are most frequently found in the mandibular lymph nodes, although generalised disease is often seen, with involvement of the lungs and thoracic lymph nodes (113). Direct contact between wild boar and other species is thought to be very rare in Mediterranean habitats and inter-species transmission of MTC involving wild boar is considered to occur indirectly at locations such as waterholes (114). Although, transmission of TB between wild boar and cattle could be minimised by culling of wild boar and preventing inter-species contact, vaccination could be a more cost-effective and sustainable disease control measure.

Oral vaccination with BCG Danish (10^6^ CFU/dose) vaccine has produced significant protection (70–80% lesion score reduction) in laboratory challenge trials (89, 115). The focus has been to vaccinate piglets as they are less likely to be infected and can be targeted by appropriate timing of bait delivery and with the use of a patented bait delivery system that reduces uptake by non-target species and excludes adult boar (116). In a recent safety study, wild boar were dosed with an oral bait containing 10^6^ CFU of BCG and groups of vaccinated animals were killed at 1, 3, 5, and 9 months post-vaccination (117). No adverse clinical signs were observed and tissues collected from the animals were culture negative for BCG. A field trial undertaken from 2012 to 2016 tested the uptake rates and efficacy of orally delivered BCG and heat-inactivated *M. bovis* vaccines in high prevalence settings (40–80% wild boar infection prevalence) in Montes de Toledo, Spain (90). The two vaccines were tested at different sites, one managed and one natural (or unmanaged) site for each vaccine, with an additional 15 non-vaccinated control sites. Vaccine baits were deployed using selected piglet feeders and the uptake rates were 50–74% in natural sites and 89–92% in managed sites. A significant reduction in the TB prevalence was only seen from one vaccinated site: heat-inactivated *M. bovis* vaccine in the managed site; with a 34% reduction in the prevalence of animals with lesions. A limitation of the study was that vaccines were deployed at different sites and efficacy was measured by the change in TB lesion prevalence compared to time zero.

### Vaccination of ferrets

In New Zealand, ferrets (*Mustela furo*) can become infected with *M. bovis* via feeding on tuberculous carcasses, particularly possums and potentially can become a source of infection for other wildlife or cattle (118). In most circumstances, ferrets are simply spill-over hosts and as yet, there is no confirmation that ferrets act as true maintenance hosts in New Zealand. Rather, ferrets could be characterised as extended spill-over hosts in which *M. bovis* infection originally acquired from possums could occasionally cycle within a ferret population before disappearing (119). Vaccination has been considered as a possible control measure for ferrets and in the first of two vaccination trials, ferrets orally vaccinated with BCG incorporated into dietary meat were partially protected against oral challenge with virulent *M. bovis* (91). In the second trial, vaccination of ferrets with BCG by the subcutaneous route resulted in reduced severity of disease following experimental infection with *M. bovis* (92).

### Vaccination of african buffalo

*M. bovis* infection is currently endemic in the Greater Kruger National Park Complex and the Hluhluwe-iMfolozi Park (120, 121), as well as in several private farms and conservancies in South Africa (122). African buffaloes are likely to be major maintenance hosts of TB (123) and play an important role in spill-over infection to other wildlife species, and of particular importance is spread of infection to predators (lions), large browsers (white rhino) and other co-located species such as kudu, baboons, and warthogs (124). In addition, there has been “spill-back” to domestic cattle (125). As “test and cull” is not a viable option for free-ranging buffaloes due to logistical impracticality and the animals' extensive geographical range, vaccination remains the only realistic alternative.

A preliminary vaccine trial was undertaken in semi-free-range buffalo to assess the efficacy of BCG vaccine. Two doses of BCG were administered subcutaneously (10^7^ CFU of BCG) and the buffaloes were challenged with virulent *M. bovis* via the intratonsilar route. The study did not reveal significant differences in the number of lesioned animals between the vaccinated and control groups (7). There were various contributing factors which could have played a role in the perceived negative results such as the age of vaccinated animals with the majority being older than 12 months at the start of the study, the route of vaccine application, challenge dose, exposure to non-tuberculous mycobacteria and stress on the animals with the grazing limitations. Future studies should aim to determine if BCG vaccination could reduce TB in vaccinated herds compared to non-vaccinated herds by targeting buffaloes <12 months old and monitoring over a period of 5–10 years in order to determine true disease status. If successful, vaccination could have a positive cascading effect, reducing *M. bovis* disease rates in other animal species. The available data does not suggest any risk to “off-target” species from BCG delivery, which reduces the ethical barriers to implementation.

## Safety of BCG vaccine in target and non-target species

BCG vaccine is one of the most widely used human vaccines, with 100 million children receiving the vaccine annually and remains one of the safest vaccines available. Reports of adverse reactions arising from BCG vaccination of children are relatively uncommon and a review of reactions to BCG vaccine in humans and animals has recently been provided by Murphy et al. (126). More severe reactions to BCG vaccine in humans were often the result of vaccination of immune-compromised individuals and factors influencing the development of adverse reactions included the potency and dose of the vaccine strain, route of delivery, age and immune status of the host and skill of the operator administering the vaccine. The most common reactions were local and regional reactions, which were generally self-limiting where suppurative lymphadenitis and abscesses were the most frequent occurring reactions.

BCG vaccine has been tested in a large number of animal species (Table 4) and relatively minor adverse clinical signs have been observed in some cases. In cattle, Francis (19) described local lesions arising following subcutaneous administration of large doses of BCG, similar to those seen with inoculation of large doses of dead bacilli, but no progressive lesions were produced and bacilli were gradually eliminated from the body. Repeat passaging of BCG vaccine in animal species is still to be undertaken to ensure no reversion to virulence, but the evidence from its use in humans for nearly a century has emphasised the safety of the vaccine.

**Table 4:** Studies of safety of BCG vaccine of different strains in target and non-target animal species.

**Animal species**	**BCG** **strain**	**Adverse clinical signs**	**Key references**
Mouse	Pasteur	Cervical lymphadentitis—oral, None—S/C	(127)
Hamster	Tice	Pleural reaction—I/P high dose	(128)
Guinea pig	Tice	None—I/D	(129)
Rabbit	Phipps	Local abscess—I/D	(130)
Dog	Tice	Mild pleural reaction—I/P	(128)
Monkey	Pasteur	Axilliary lymphadenitis- high dose I/D	(131)
		Local draining abscess—medium dose S/C	
Sheep	Danish	None—S/C	(70)
Horse	Pasteur	None—intralesion injection	(132)
Goat	Danish	None—S/C, no shedding in milk or faeces	(65)
Cattle	Pasteur	Local swelling at injection site—S/C	(19)
White-tailed deer	Danish	None—S/C, Oral, BCG persisted in draining LNs (12 months), transmission to in-contacts	(79, 80)
Red deer	Pasteur	None—S/C, persisted in draining LNs (14 wks)	(133)
Possum	Danish	None—Oral, shedding in faeces (1 wk), persisted in mesenteric LNs (8 wks)	(99)
	Pasteur	None—Aerosol, S/C	(93)
Badger	Danish	None—Oral, single and repeat dosing, shedding in faeces (48 h)	(109)
	Danish	I/Mus, S/C, single, and repeat injection, local swelling at injection site	(106)
Wild boar	Danish	None—low dose oral, BCG not re-isolated	(117)
Ferret	Pasteur	None—Oral, S/C	(91, 92)
African buffalo	Pasteur	None—S/C	(7)

*Vaccination route: I/D, intradermal; I/M, Intramuscular; I/P, Intrapleural; S/C, Subcutaneous*.

Local abscesses or nodules have been observed following subcutaneous injections of BCG in a number of other animal species and these resolved relatively quickly (Table 4). No adverse effects have been observed after oral administration of BCG in animals other than cervical lymphadenitis observed in mice (127), similar to a reaction observed occasionally in young vaccinated children (126). Following oral dosing with BCG of possums and badgers, transient shedding of low numbers of BCG in faeces was observed (99, 109). Transmission of BCG from vaccinated animals to in-contact non-vaccinates has only been recorded in white-tailed deer (79). There is a risk that distribution of oral baits containing BCG for wildlife could lead to uptake by non-target animal species such as cattle, resulting in a subsequent positive tuberculin skin test response and therefore special care with regards to bait distribution is essential. The chance of cattle becoming infected with BCG from faecal contamination of pasture or feed from vaccinated wildlife would be very rare as tuberculin skin test reactivity following oral administration of BCG to cattle has only been recorded with high doses of BCG (≥10^7^ CFU) (34). Similar to the situation in humans, BCG vaccine is considered to be a safe vaccine in all animal species tested.

## Conclusions

Experimental challenge studies in domestic livestock including cattle, goats, sheep and farmed deer have demonstrated that BCG vaccination can moderate the severity of the disease, while field trials in cattle and goats have indicated that vaccination can also reduce infection. No single vaccine has been shown to be better than BCG in cattle, although combinations of BCG with various subunit TB vaccines have produced encouraging results and could have application in the future [reviewed in (2, 134)]. Vaccination of cattle with BCG would have greatest application in countries where “test and slaughter” strategies are not affordable or socially acceptable and in this situation, BCG could play a role in reducing the spread of bovine TB. It is well-recognised in humans that BCG confers some non-specific protective effects against other pathogens (135), but this has yet to be evaluated in cattle. Improvement in general health of animals *per se* and/or increased productivity post-BCG vaccination could potentially have benefits in developing countries. Strategic use of BCG vaccine for livestock could also be implemented in regions where wildlife serve as reservoirs of infection, particularly where it is not feasible to contain the spread of infection from wildlife. In these situations, DIVA tests, particularly skin tests utilising specific *M. bovis* antigens, could be used in livestock in association with vaccination to allow vaccination to be integrated with “test and slaughter” control measures.

A number of recommendations can be made from the experimental challenge and field experiments in cattle. Calves should be vaccinated with BCG as young as possible, optimally by 2–4 weeks of age, at doses of 10^5^-10^6^ CFU parenterally or 10^8^ CFU orally and no differences have been detected in protection induced by two of the most commonly used BCG strains, Pasteur and Danish. Protection has been shown to wane between 1 and 2 years post-vaccination and revaccination is recommended every 1–2 years to maintain levels of immunity. BCG vaccine has been shown to be safe in cattle and vaccination of cattle pre-infected with *M. bovis* is not likely to exacerbate or cure infection. Vaccination is likely to produce false reactions in traditional TB diagnostic tests in the first 12 months post-vaccination and as protection induced by BCG is not complete, DIVA tests should be used if “test and slaughter” control policies are in place. It would be preferable to use BCG vaccination in association with other TB control measures such as minimising the chance of early exposure to *M. bovis* by feeding young calves with colostrum or milk from non-reactor cattle or with heated milk, segregating reactor and non-reactor cattle into separate herds and keeping vaccinated calves with the non-reactor animals.

The field testing of BCG vaccine in possums and badgers administered via oral or parenteral routes have resulted in the induction of significant reductions in infection of these animals and a parenteral BCG vaccine has now been licensed for use in badgers in the UK. In wild boar, feral deer and ferrets, BCG vaccine has been shown to induce significant levels of protection against experimental challenge with TB. Practical systems for delivery of oral bait TB vaccines to wildlife have now been established, but further research is necessary to improve oral bait formulations with appropriate attractants, systems for optimising bait distribution and avoiding bait uptake by non-target species. BCG vaccine has been shown to be safe in all animal species tested, although BCG has been isolated from lymph nodes draining vaccination sites and from faeces of animals for a short period following oral vaccination. There was evidence that vaccinated white-tailed deer could transmit BCG to non-vaccinated pen-mates, but not to cattle exposed to the room previously occupied by the vaccinated deer.

In summary, there have been major advances in the past 10–20 years in our understanding of the factors influencing BCG vaccine efficacy for domestic livestock and wild animals. To optimise the use of BCG vaccine, it will be important to continue to field test BCG vaccine in the various animal species in different environments, husbandry systems and in the presence of varying levels of disease prevalence as well as evaluating the practical application of DIVA tests. Although BCG vaccine may not provide complete protection against exposure to *M. bovis*, the protection should be sufficient to markedly reduce onward transmission to others animals. This feature could ensure that BCG vaccine could be particularly valuable for reducing infection in wildlife populations and in domestic animals where infection is currently very high and where “test and slaughter” control strategies are not able to be undertaken. There are numerous technical hurdles still to be overcome before an economically viable oral vaccine for use in badgers in the UK might be available. In the meantime it is beholden on stakeholders to make the best use of the existing tools available, this includes the intramuscular BadgerBCG vaccine. Cattle BCG vaccination in countries using “test and slaughter” control strategies also face significant hurdles. For example, a BCG vaccination-compatible DIVA test needs to be validated to allow vaccination to continue alongside traditional “test and slaughter” control programmes; currently BCG vaccination is prohibited under EU and some other countries' legislation and this would need to change; finally, cost-benefit analyses would decide whether deployment would proceed.

## Author contributions

BB, HV, MC, and L-MdK-L wrote sections of the manuscript. All authors contributed to the manuscript revision, read and approved the submitted version.

### Conflict of interest statement

The authors declare that the research was conducted in the absence of any commercial or financial relationships that could be construed as a potential conflict of interest.
